# Mosaic Loss-of-Y Chromosome in Men Is Associated With Aortic Enlargement But Not Aortic Stenosis

**DOI:** 10.1016/j.jacadv.2026.102911

**Published:** 2026-06-17

**Authors:** Matteo Morello, Jesse D. Cochran, Bethany A. Gholson, Christopher R. Xie, Weiting Huang, Sahithi Mikkilineni, Skyler Nelson, Coleen McNamara, Kenneth Walsh, Jonathan R. Lindner

**Affiliations:** a Cardiovascular Division, University of Virginia, Charlottesville, Virginia, USA; b Robert M. Berne Cardiovascular Research Center, University of Virginia, Charlottesville, Virginia, USA; c Department of Molecular and Translational Medicine, University of Brescia, Brescia, Italy; d Beirne Carter Immunology Center, University of Virginia, Charlottesville, Virginia, USA

**Keywords:** aortic aneurysm, aortic stenosis, loss-of-Y chromosome

Degenerative aortic stenosis (AS) and thoracic aortic aneurysm (TAA) share pathogenic pathways that involve proinflammatory signaling, transforming growth factor (TGF)-β1/SMAD signaling, and myofibroblastic or osteogenic transformation of resident cells.^1^^,^^2^ There is also a male predominance for AS and development of TAA at earlier age. Mosaic loss-of-Y (LOY) chromosome is a phenomenon that occurs with aging in males and has been associated with age-related diseases through enhanced TGF-β signaling pathways.^3^ Given the pathogenic role of TGF-β1/SMAD in AS and TAA, together with recent data indicating a role of Y-linked pathways in regulating valve calcification,^4^ we hypothesized that LOY in circulating blood cells is associated with either the development of AS or enlargement of the ascending aorta at an early age.

This cross-sectional study was approved by the Investigational Review Board at the University of Virginia. Peripheral blood was obtained from 64 men with moderate or greater degenerative AS (nonbicuspid) confirmed by echocardiography, and 69 men without significant valvular heart disease matched for age. Allele fraction for LOY was measured in DNA isolated from cryopreserved peripheral blood mononuclear cells. Taqman/digital polymerase chain reaction assay was employed to estimate the relative number of copies of AMELY and AMELX, serving as proxies for the number of Y and X chromosomes, respectively. 2′-chloro-7′-phenyl-1,4-dichloro-6-carboxy-fluorescein-tagged probes were used to target a 6-bp difference between AMELY and AMELX. Quantitative echocardiographic data for our analysis were reviewed and reanalyzed by an Advanced Cardiac Sonographer according to guidelines and largest aortic dimension (sinuses and immediately beyond the sinotubular junction) was recorded. Group comparisons were performed with Student’s t-test or Mann-Whitney U test, as appropriate. Bivariate associations were evaluated using Pearson or Spearman coefficients, as appropriate, whereas multivariable linear regression was subsequently performed after residual diagnostics confirmed no major assumption violations. Nonlinear associations between LOY and echocardiographic data were explored using natural cubic splines.

Subjects with greater LOY had a larger maximum aortic root dimension indexed to body surface area than those with less LOY, particularly when using a cutoff of 6% of leukocytes which was selected based on 75th percentile of the entire distribution (Figure 1A). The proportion of men with any degree of aortic enlargement, defined as ≥1.8 cm/m^2^ was significantly greater in those with LOY ≥6% (54.2% vs 16.0%; chi-square *P* = 0.002). There was a significant correlation between age and ascending aortic dimension in those with low LOY (r = 0.35; *P* = 0.002). This correlation was lost in subjects with high LOY (r = 0.01; p-0.94) due to larger aortic dimension earlier in age. Treating LOY as a continuous variable, a restricted cubic spline model suggested an overall association between LOY and indexed aortic dimension on echocardiography (Figure 1B). However, formal comparison between linear and spline-based models did not support a significant nonlinear component. In univariable linear regression, LOY was positively associated with indexed aortic dimension (β [95% CI] 0.30 [0.11-0.47]; *P* = 0.002). Multivariable linear regression analysis indicated that this association remained significant – although attenuated - (b [95% CI] 0.01 [0.0001-0.02]; *P* = 0.04) after controlling for covariates including age, hypertension, aortic valve area, diabetes, smoking, and chronic renal insufficiency. Aortic dimension was slightly greater in those with vs without AS (3.56 ± 0.31 vs 3.36 ± 0.31 cm; *P* = 0.001), although relation between aortic valve area and aortic dimension was not significant in the multivariable model (*P* = 0.22).

**Figure 1:**
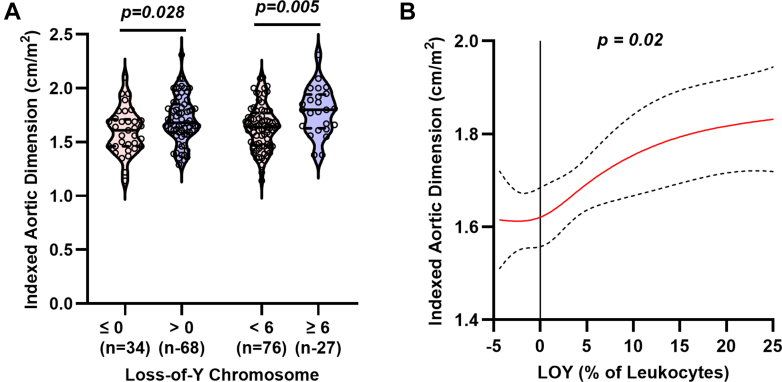
Link Between Mosaic Loss-of-Y Chromosome and Thoracic Aortic Enlargement or Aortic Stenosis in Men (A) Violin plots (solid line = median, dashed line = IQR) showing the maximal thoracic aortic dimension at the root indexed to body size according to LOY status defined by greater than or equal to zero percent (with 33 subjects having LOY <0), or ≥ 6%. (B) Cubic spline plot with 95% CIs showing relation between LOY and indexed thoracic aortic dimension. LOY = Loss-of-Y.

Unlike the case with aortic root dimension, the degree of AS, quantified by the aortic valve area indexed to body surface area, was not different according to LOY status irrespective of the threshold applied (*P* = 0.511 for LOY defined as >0%; *P* = 0.91 for LOY defined as ≥6%). The degree of LOY was not different in controls vs patients with AS (median LOY [95% CI]: 1.08% [0.20-2.64] vs 2.56% [0.63-4.71]; *P* = 0.321). In propensity-score–matched analyses (age, diabetes, and hypertension), age remained positively correlated with LOY in both AS and control cohorts. Notably, there was no difference in the age-LOY relationship in patients with vs without AS, supporting the view that age-related AS progression occurs largely independent of LOY. Treating LOY as a continuous variable, a restricted cubic spline model showed no significant association between LOY and indexed aortic valve area (*P* = 0.45).

Mosaic LOY is a common somatic mutation in males and is increasingly recognized as a contributor to age-related diseases and shortened lifespans in males. Analysis of the UK Biobank data has linked LOY to increased mortality from hypertensive heart disease, heart failure, and aortic disease in men.^3^ Given the recognized pathogenic role of TGF-β1 in LOY-related diseases,^3^ and potential role of Y-linked osteogenic regulation in the stimulation of valve interstitial cells,^4^ we sought to further delineate the relationship between hematopoietic LOY and diseases of the aorta and aortic valve. Moreover, a prospective study from the UK biobank has linked LOY with a reported new-onset diagnosis of valvular heart disease, although there was no quantitative imaging data and the LOY threshold (40%) was uncommon and high owing to low-sensitivity of the LOY assay used.^5^ Our cross-sectional analysis revealed an association between hematopoietic LOY with the dimension of the thoracic aortic root, but no apparent link with aortic valve area or the diagnosis of AS. After controlling for age as an independent variable, we found that a greater degree of LOY may be an important factor driving accelerated aortic enlargement in men. Whether this process involves TGF-β1 signaling from platelets, a rich source of TGF-β1 that likely contribute to AS and aortic remodeling or other cell types including resident mural cells is unknown. A limitation of the study is the cross-sectional design which limits our ability to state that LOY is a risk factor or contributor to TAA or AS. In addition, we did not design our study to compare men with vs without TAA. Nevertheless, we did find that the prevalence of aortic enlargement was more than 3-fold higher in the highest quartile for LOY (>6%). Taken together, these findings support the need for further clinical validation in larger cohorts to establish the relationship between LOY and the development of clinically significant TAA. They also highlight the importance of future mechanistic studies leveraging mouse models to determine whether hematopoietic LOY plays a causal role in aortic dilation.

## Funding support and author disclosures

Dr Lindner is supported by grants R01-HL171377, R01-HL078610, and R01-HL165422, from the United States 10.13039/100000002National Institutes of Health (NIH), Bethesda, MD; and grant 18-18HCFBP_2 to 0009 from NASA. Dr Walsh is supported by 10.13039/100000002NIH grants R01-AG092528 and R01-AG086508. Dr Cochrane is supported by a Wagner Fellowship and iPrime Student to Fellowship Award from UVA. All other authors have reported that they have no relationships relevant to the contents of this paper to disclose.
